# Supplementary material for: WordCommentsAnalyzer: A windows software tool for qualitative research
Source: F1000Res. 2018 Sep 4;7:536. Originally published 2018 May 3. [Version 2] doi: 10.12688/f1000research.14819.2 (PMC6137411; doi:10.12688/f1000research.14819.2)
Supplement: Supplementary file 3 [file f1000research-7-17614-s0002.tgz › b2a79496-4ada-47c4-9682-a99ce867836d.zip!/Eight Iranian Health Journals/Journal of Research in Rehabilitation Sciences _24.docx]

© Journal of Research in Rehabilitation Sciences . This work is licensed under CC BY-NC 4.0 (https://creativecommons.org/licenses/by-nc/4.0/).

Volume & Issue: دوره 10، شماره 1: 1393

Title: مقایسه اثر درمانی تکنیک فشار ایسکمیک بر روی نقاط ماشه ای عضله تراپزیوس فوقانی در وضعیت طبیعی و وضعیت کشش پسیو عضله در کاربران کامپیوتر

http://jrrs.mui.ac.ir/index.php/jrrs/article/view/1322

چکیده مقدمه: به عقيده ي تعدادي از صاحبنظران سندرم درد مايوفاسيا علت اصلي درد گردن و شانه مي باشد كه با نقاط ماشه اي همراه مشخص مي شود. هدف از اين مطالعه، بررسی اثر درمانی فشار ایسکمیک بر روی نقاط ماشه ای عضله ی تراپز فوقانی در وضعیت طبیعی و وضعیت کشش عضله بود. مواد و روش ها: در این مطالعه 60 نفر از کاربران کامپیوتر که دارای نقاط ماشه ای در عضله ی تراپز فوقانی بودند به طور تصادفی به دو گروه تقسیم شدند :  گروه اول تکنیک فشار ایسکمیک را در وضعیت کشش عضله و گروه دیگراین تکنیک را در وضعیت طبیعی عضله دریافت کردند. شدت درد ، میزان دامنه حرکتی فعال فلکسیون جانبی گردن و آستانه فشار درد قبل از مداخله اندازه گیری شد . 5 دقیقه پس از درمان و 3 روز پس از درمان نیز اندازه گیری های فوق تکرار شد . روش های آماری مورد استفاده آزمون کولموگروف - اسمیرنوف ، آزمون آنالیز واریانس با اندازه های تکراری ،آزمون تی مستقل و آزمون مجذور کای بود. یافته ها: نتایج، بهبودی معنیداری را از نظر  متغیرهای شدت درد ، دامنه حرکتی فعال فلکسیون جانبی گردن و آستانه فشار درد بعد از مداخله در هر دو گروه نشان داد(05/0 P< ).از نظر متغیرهای مورد مطالعه بین دو گروه اختلاف معنی داری مشاهده نشد. نتیجه گیری : تکنیک فشار ایسکمیک در وضعیت کشش عضله و وضعیت طبیعی عضله در کاهش شدت درد ، افزیش دامنه حرکتی فعال فلکسیون جانبی گردن و آستانه فشار درد موثر بود . بطورکلی  گروهی که تکنیک فشار ایسکمیک را در وضعیت کشش عضله دریافت کرده بودند بهبود بیشتری را از نظر هرسه متغیر نشان دادند ،هرچند تفاوت میان دو گروه از نظر آماری معنی دار نبود.

Title: مقايسه يادگيري حرکتي صريح و یادگیری حرکتی ضمني توالي حرکتي در کودکان مبتلا به اختلال کمبود توجه و بيش فعالي با همتايان عادي

http://jrrs.mui.ac.ir/index.php/jrrs/article/view/1172

مقدمه: هدف از مطالعه حاضر، بررسي دو نوع يادگيري حرکتي صريح و ضمني در گروه کودکان مبتلا به اختلال کمبود توجه و بيش فعال و همتايان عادي، با استفاده از ابزار زمان عکس العمل متوالي است. مواد و روش ها: حجم نمونه  برای هر گروه برابر 12 در نظر گرفته شد.  در اين پژوهش، نمونه ها شامل 24کودک مبتلا به اختلال کمبود توجه و بیش فعالی در دو گروه یادگیری صریح و یادگیری ضمنی و 24 کودک همتای عادی  در دو گروه یادگیری صریح و ضمنی  جای گرفتند. آزمون هاي آماري تحليل واريانس با اندازه گيري مکرر براي اندازه گيري زمان واکنش و خطاي پاسخ، و تي زوجی براي مقايسه داده هاي توالي هاي منظم و نامنظم در هر گروه مورد استفاده قرار گرفت. يافته ها: مقايسه اين دو نوع يادگيري در سرعت (زمان واکنش) و دقت (تعداد خطاي پاسخ) نشان دهنده اين بود که: در گروه يادگيري حرکتي صريح کودکان اختلال کمبود توجه وبيش فعال،تعداد خطاها (/0120 (P-value=و زمان واکنش (/0120 (P-value= بود. و در گروه يادگيري حرکتي ضمني کودکان اختلال کمبود توجه وبيش فعال، تعداد خطاها (/0940 (P-value=و زمان واکنش (/9540 (P-value= بود. در گروه صريح همتايان عادي، تعداد خطاها (/0080 (P-value=و زمان واکنش (/050 (P-value= بود و گروه ضمني همتايان عادي ، تعداد خطاها (/0110 (P-value=و زمان واکنش (/4420 (P-value= بود. نتيجه گيري: نتايج اين تحقيق نشان داد که توضيح و تشريح روند تکليف، در آموزش توالي حرکتي کودکان اختلال کمبود توجه و بيش فعال اثر بخشي بيشتري دارد. لذا آموزش هاي قبل از تمرين بايد در برنامه اين کودکان قرار بگيرد.

Title: بررسی اثربخشی رویکرد یکپارچگی حسی در درمان اختلال آپراکسی ساختاری کودکان اتیسم بالای چهار سال شهر شیراز: یک مطالعه مقدماتی

http://jrrs.mui.ac.ir/index.php/jrrs/article/view/1289

عنوان: بررسی اثربخشی رویکرد یکپارچگی حسی در درمان اختلال آپراکسی ساختاری کودکان اتیسم بالای چهار سال شهر شیراز: یک مطالعه مقدماتی مقدمه: آپراکسی یکی از اختلالات مطرح در کودکان اتیسم است که همبستگی زیادی با آسیبهای رفتاری و ارتباطی- اجتماعی این کودکان دارد. از میان انواع مختلف آپراکسی یکی از شایع ترین آنها آپراکسی ساختاری است. از آنجایی که رویکرد یکپارچگی حسی به عنوان یکی از درمانهای مرسوم برای این کودکان است لذا با توجه به مناقشه های موجود در خصوص کارآیی این رویکرد مطالعه ی حاضر به هدف بررسی اثربخشی رویکرد یکپارچگی حسی در درمان آپراکسی ساختاری کودکان اتیسم طراحی گردید. مواد و روش ها: این مطالعه از نوع شبه تجربی می باشد و در یکی از مدارس اتیسم شهر شیراز در تابستان 1391 انجام شد. نمونه گیری به شیوه آسان از میان کودکان اتیسمی که خانواده هایشان مایل به همکاری بودند انجام شد. از میان 21 کودک واجد شرایط 11 نفر بطور تصادفی به گروه کنترل و 10 نفر به گروه مداخله اختصاص یافتند. کودکان گروه مداخله به مدت 20 جلسه تحت درمان با رویکرد یکپارچگی حسی قرار گرفتند و کودکان گروه کنترل هیچ گونه درمانی دریافت نکردند. برای اندازه گیری متغیر آپراکسی ساختاری کودکان از آزمون محقق ساخته Block test استفاده شد. آزمون ANCOVA برای ارزیابی نتایج استفاده شد. یافته ها: نتایج آماری تفاوت معناداری را در بهبود آپراکسی ساختاری بین دو گروه کنترل و مداخله نشان نداد (0.2005 =F ، 0.175 =P). نتیجه گیری: از آنجایی که استفاده از رویکرد یکپارچگی حسی از اقبال زیادی در میان کاردرمانگران برخوردار است لذا با توجه به یافته ی این مطالعه توصیه می گردد چنانچه هدف درمانی برای کودک اتیسمی کاهش اختلال آپراکسی ساختاری کودک است علاوه بر استفاده از رویکرد یکپارچگی حسی از سایر رویکردهای درمانی نیز در خلال درمان استفاده شود تا از این طریق بتوان شانس اثر بخشی فرآیند درمان را بالا برد.

Title: بررسي روايي محتوايي نسخه فارسي «ليست ارزشيابي ناتواني کودکان(PEDI)

http://jrrs.mui.ac.ir/index.php/jrrs/article/view/1360

مقدمه: با توجه به تغییر رویکرد در توانبخشی کودکان با ناتوانی، کارکرد یکی از ویژگی های موثر در کیفیت زندگی این کودکان است. «ليست ارزشيابي ناتواني کودکان» به منظور ارزیابی کارکرد در کودکان 6 ماه تا 5/7 سال طراحی شد. هدف از اين مطالعه ترجمه «ليست ارزشيابي ناتواني کودکان» به فارسي و بررسي روايي محتوايي نسخه فارسي آن  است. مواد و روش­ ها: فرآيند ترجمه بر اساس پروژه بين­المللي کيفيت زندگي انجام شد. تهیه نسخه فارسي «ليست ارزشيابي ناتواني کودکان» طی 3 مرحله انجام شد: ترجمه (ترجمه از انگلیسی به فارسی و برگردان دوباره به انگلیسی، توسط 4 مترجم)، بررسي کيفيت ترجمه (توسط 8 متخصص توانبخشیِ مسلط به زبان انگليسي) و بررسي روايي محتوايي آن (توسط 15 متخصص توانبخشی کودکان). یافته ­ها: پاسخ­دهندگان ترجمه فارسی «ليست ارزشيابي ناتواني کودکان» را قابل قبول دانستند. روایی محتوایی ترجمه فارسی  «ليست ارزشيابي ناتواني کودکان» نیز بدون تغییر در محتوای آن به تایید رسید. نتیجه گیری: نتايج نشان مي­دهد که ترجمه فارسي«ليست ارزشيابي ناتواني کودکان» قابل قبول و دارای روایی محتوایی است.

Title: بررسی نتایج غربالگری شنوایی همگانی نوزادان با استفاده از پروتکل های TEOAE-TEOAEو TEOAE

http://jrrs.mui.ac.ir/index.php/jrrs/article/view/1333

مقدمه: افت شنوایی دایمی، یکی از شایعترین اختلالات مادرزادی با شیوع 1 تا 3 در 1000 تولد گزارش شده است. بهترین راه تشخیص زود هنگام این مشکل، غربالگری همگانی شنوایی می باشد. این مطالعه، به بررسی تاریخچه غربالگری و میزان شیوع کم شنوایی در بین نوزادان متولد شده و نتایج به دست آمده از پروتکل های مختلف غربالگری شنوایی در شهر ورامین می پردازد. مواد و روش ها: این مطالعه به صورت توصیفی- مقطعی بر روی 29476 نوزاد متولد شده در شهر ورامین از دی ماه سال 1380 تا پایان سال 1391 صورت گرفت. از سال 1380 تا 1390 پروتکل TEOAE-TEOAE و از سال 1390 تا 1391  پروتکلTEOAE-AABR انجام شد. درصورت منفی بودن آزمایش در دو مرحله، ارزیابی های تشخیصیABR  و ارجاع به متخصص گوش، حلق و بینی انجام پذیرفت و پس از تشخیص قطعی در صورت نیاز، مداخلات توانبخشی صورت گرفت. یافته ها: شیوع میزان کم شنوایی در اولین پروتکل 6/1 در 1000 تولد  و در دومین پروتکل 2 در 1000 تولد گزارش شود. 55 درصد نوزادان مبتلا به افت شنوایی دارای عوامل خطر و 45 درصد موارد مبتلا به افت شنوایی فاقد هرگونه عامل خطر بودند. نتیجه گیری: با توجه به آمار به دست آمده از شیوع کم شنوایی در نوزدان بدون عوامل خطر، انجام آن برای همه الزامی بوده و از آن جا که پاسخ های مثبت کاذب نگرانی های بسیاری برای خانواده ها به بار می آورد، جهت حمایت از آن ها سعی در هر چه بهتر شدن پروتکل های غربالگری نماییم.

Title: تاثیر تمرینات منتخب ثبات دهنده مرکزی بر درد و ناتوانی عملکردی بیماران مبتلا به کمردرد مزمن غیر¬اختصاصی

http://jrrs.mui.ac.ir/index.php/jrrs/article/view/1654

مقدمه: کمردرد یا درد ستون فقرات شایع­ترین عارضه عضلانی-استخوانی است. روش­های متعددی برای درمان کمردرد وجود دارد ولی دستیابی به روشی مختصر، ساده و فشرده نیاز امروز جوامع صنعتی است. هدف از انجام این تحقیق بررسی تاثیر شش هفته تمرینات منتخب ثبات­دهنده مرکزی بر درد و ناتوانی عملکردی بیماران مبتلا به کمردرد مزمن غیراختصاصی بود. مواد و روش­ها: بیست بیمار مبتلا به کمردرد مزمن غیراختصاصی (10 زن و 10 مرد) با میانگین سنی4/3 ± 55/24 سال، میانگین قد 6/9 ± 15/164 و میانگین وزن04/5 ± 25/65  به مدت شش هفته برنامه تمرینی منتخب ثبات­دهنده را زیر­نظر فیزیوتراپیست انجام دادند. میزان درد با استفاده از مقیاس دیداری درد (VAS) و میزان ناتوانی عملکردی با استفاده از Oswestry Disability Index قبل و پس از شش هفته مداخله تمرینی اندازه­گیری شد. نرمال بودن داده­ها با استفاده از  Shapiro–Wilk Testو میزان تغییرات درون­گروهی با استفاده از Dependent t-test محاسبه شد (05/0p<). همچنین تجزیه و تحلیل داده­ها با استفاده از نرم­افزار SPSS21 انجام شد. یافته­ها: نتایج نشان­دهنده کاهش معنی­دار در میزان درد، از 13/2 ±12/7 به 67/2 ± 75/3 در بیماران مبتلا به کمردرد مزمن غیراختصاصی پس از شش هفته تمرین ثبات­دهنده منتخب بود (00/0 =p). همچنین نتایج بدست آمده از ODI نشان­دهنده بهبودی معنی­دار در میزان ناتوانی عملکردی پس از انجام شش هفته مداخله تمرینی بود و از 42/15 ± 8/40 در پیش آزمون به 52/14 ±3/23 در پس­آزمون کاهش یافت (00/0 =p ). نتیجه­گیری: با توجه به نتایج تحقیق حاضر، تمرینات منتخب ثبات­دهنده با کاهش زمان هر جلسه تمرینی می­تواند در توانبخشی بیماران مبتلا به کمردرد مزمن نقش به سزایی داشته باشد و باعث استفاده مداوم از آن­ها در طول زندگی شود، لذا می­توان از این پروتکل به عنوان جایگزینی برای تمرینات پرحجم و زمان­بر استفاده کرد.

Title: بررسی پایایی بین دو آزمونگرAction Research Arm Testو قسمت اندام فوقانی آزمون فوگل مایر در افراد با سكته مغزي شهر قزوين

http://jrrs.mui.ac.ir/index.php/jrrs/article/view/1141

مقدمه:سکته مغزی یکی از شایع ترین بیماریهای نورولوژیک تهدید کننده زندگی و مهم ترین علت ناتوانی افراد مسن است. بروز آسیب حرکتی در یک اندام فوقانی به عنوان  اصلی ترین عامل وابستگی در فعالیتهای روزمره زندگی به شمار می آید.Action Research Arm Test وقسمت اندام فوقانی آزمون فوگل مایر به منظور ارزیابی عملکرد حرکتی اندام فوقانی بیماران همی پلژی تهیه شده است.هدف این پژوهش بررسی پایایی بین دو آزمونگر این دو آزمون بود. روش بررسی: در این پژوهش که از نوع تحلیلی و اعتبار سنجی می باشد، پس از تهیه وسایل مورد نیاز برای انجام آزمون،آزمونها بر روی 20 نفر از بیماران سکته مغزی 65-40 سال که به شیوه در دسترس از کلینیکهای کاردرمانی و مراکز توانبخشی استان قزوین مطابق معیارهای لازم انتخاب شده بودند توسط دو کاردرمانگر در یک جلسه اجرا گردید.به منظور بررسی پایایی بین دو آزمونگر،ازمونها از روش آماری ضریب همبستگی درون  گروهی(ICC) استفاده شد، از نرم افزارSPSS18 برای آنالیز داده ها استفاده شد و سطح معنی داری 05/0  در نظر گرفته شد. یافته ها: آزمون ICC که به منظور بررسی پایایی بین دو آزمونگر به کار رفته بود محاسبه و برابر 972/0ARATو 977/0 آزمون فوگل مایربه دست آمد.(05/0P<) نتیجه گیری:ARATو آزمون فوگل مایر از پایایی بین دو آزمونگر  قابل قبولی برای استفاده در بیماران سکته مغزی برخوردار است.

Title: شناسایی و تعیین تأثیر برخی ریسک فاکتورهای درونی و برونی بر میزان شیوع آسیب لیگامنت متقاطع قدامی فوتبالیست های حرفه ای دنیا

http://jrrs.mui.ac.ir/index.php/jrrs/article/view/1256

مقدمه: پارگی لیگامنت متقاطع قدامی یکی از متداولترین آسیبها در فوتبالیستها می باشد، لذا هدف از تحقیق حاضر شناسایی تأثیر برخی ریسک فاکتورهای درونی وبرونی برمیزان شیوع آسیب لیگـامنـت متقاطـع قـدامـی فوتبالیستهای حرفه ای دنیا بود. موادوروش ها: تعداد 100فوتبالیست حرفـه ای(ســن12/4±05/25سال، قد06/0±81/1متر) از چندکشوردنیا که دچار آسیب لیگامنت متقاطع قدامی شده بودند،به عنوان نمونه های تحقیق درنظر گرفته شدند. اطلاعات مربوط به ریسک فاکتورهای درونی(پای برتر، قد، سن) وبرونی(پست، بازیهای خانگی و خارج از خانه،زمانهای مختلف فصل،مسابقه و تمرین)ازطریق بررسی پرونده های پزشکی،مصاحبه باپزشک تیم و همچنین از دیتابیس های معتبراینترنتی جمع آوری شد.برای آنالیزداده ها ازآزمون خی دو باسطح معناداری(05/0>P) استفاده شد. یافته ها: نتایج نشان داد که شیوع آسیب لیگامنت صلیبی قدامی بین پستهای مختلف بطور معناداری (P<0/05) متفاوت بود،بطوری که هافبکها بیشترین(%38)و دروازه بانها کمترین(%6)تعداد آسیب را داشتند. میزان وقوع آسیب در پای برتر(%52)بیشتر از پای غیربرتر(%48)بود،اما این تفاوت از لحاظ آماری معنادار نبود(P>0/05). بازیکنان با قد83/1-1/79متر بیشترین میزان آسیب(%26)و بازیکنان با قــد73/1-69/1مترکمترین میزان آسیب(%13)را داشتند. بین5گروه قدموردنظر تفاوت معناداری یافت نشد(P>0/05). آسیبهای رخ داده در بازیهای خارج از خانه (%6/50)بطورغیرمعناداری(P>0/05)بیشتر ازبازیهای خانگی(%4/49)بود. در اوایل فصل مسابقه این آسیب(%34) بطورمعناداری(P<0/05)بیشتر از سایر زمانها بود. تفاوت بین سنین مختلف در رابطه با آسیب این لیگامنت معـنادار بود(P<0/05)،بطوریکه بازیکنان در سن27-22سالگی تعداد آسیب(%50)بیشتری را به خود اختصاص دادند و در سن 39-34سال کمترین(%2)تعداد آسیـب رخ داد. آسیـبهای زمـان مسابـقه(%85)بطـور معنـاداری بیشـتر از زمـان تمـریـن(%15)بود(P<0/05).  نتیجه گیری: بازیکنان هافبک در سن27-22سال در اوایل فصل، بویژه در مسابقه در معرض آسیب لیگامنت متقاطع قدامی هستند. پای برتر، قد،بازی خانگی وخارج از خانه از نظر آماری بطور معناداری بر میزان شیوع این آسیب تأثیر نداشت. لذا مربی، بازیکنان وکادر پزشکی باید به این نکات مهم برای طراحی برنامه پیشگیری توجه کنند تا آسیب لیگامنت متقاطع قدامی کمتر شود.

Title: مقایسه کیفیت زندگی مادران کودکان فلج مغزی بر اساس توانایی راه رفتن کودکان

http://jrrs.mui.ac.ir/index.php/jrrs/article/view/1147

چکیده: مقدمه: مطالعات بسیاری کاهش کیفیت زندگی مادران کودکان فلج مغزی را تحت تأثیرفعالیت‌های مراقبتی نشان داده‌اند اما بررسی ارتباط کاهش کیفیت زندگی این مادران با عوامل وابسته به کودک نظیر توانایی راه رفتن کودک فلج مغزی کمتر مورد توجه پژوهشگران قرار گرفته است؛ بنابراین هدف از مطالعه حاضر بررسی تأثیر توانایی راه رفتن کودکان فلج مغزی بر کیفیت زندگی مادران آن‌هابود. مواد و روش‌ها: این مطالعه‌ی علی-مقایسه ای در سال 1390 در شهر تهران انجام شد که در آن 60 مادر دارای یک کودک فلج مغزی 4 تا 12 ساله در مطالعه شرکت کردند. جهت ارزیابی کیفیت زندگی از پرسشنامه SF-36 استفاده شد و ارزیابی سطح 1 تا 5 مقیاس GMFCS کودکان فلج مغزی توسط کار درمانگر انجام شد. ارتباط میان متغیر از طریق آزمون همبستگی پیرسون و رگرسیون خطی با کمک نرم‌افزارSPSS نسخه 16 بررسی شد. یافته‌ها: 24 مادر دارای کودک فاقد توانایی راه رفتن و 36 مادر کودک با توانایی راه رفتن در مطالعه شرکت کردند که میانگین و انحراف معیار سن بر حسب سال در مادران 02/6±79/33 و در کودکان 71/2± 11/ 7 بود. در نتیجه آنالیز داده‌ها دو گروه مادران نمره کلی کیفیت زندگی و ابعاد عملکرد اجتماعی، عملکرد جسمانی و سلامت عمومی با هم تفاوت معنادار داشتند (05/0 P<). نتیجه‌گیری: مادران دارای کودکان فاقد توانایی راه رفتن مدت زمانی بیشتری را به مراقبت از کودک خود اختصاص می‌دادند و کیفیت زندگی پایین تری را نیز گزارش کردند.

Title: شبیه¬سازی انسداد هدایت عصب بوسیله اعمال جریان متناوب فرکانس بالا با شکل موج مربعی نامتقارن و بررسی مکانیزم اثر آن

http://jrrs.mui.ac.ir/index.php/jrrs/article/view/1302

مقدمه: استفاده از جریان­های متناوب فرکانس بالا روشی ایمن، بازگشت­پذیر و دارای خاصیت انتخابی برای انسداد الکتریکی عصب و جلوگیری از انتشار پالس­های عصبی است که در دهه گذشته جهت درمان بیماری­ و ناتوانی­های مرتبط با پالس­های عصبی ناخواسته، مورد توجه محققین قرار گرفته است. با این حال کاهش دامنه تحریک، از بین بردن پاسخ هجومی اولیه و بهینه­سازی پارامترهای تحریک برای کاربردهای مختلف همچنان مورد بررسی قرار دارد. در این مطالعه با شبیه­سازی انسداد فیبرهای عصبی، کارآیی شکل­موج پیشنهادی دوفاز نامتقارن در القای بلوک عصبی و مکانیزم اثر آن مورد ارزیابی قرار گرفت. مواد و روشها: شبیه­سازی به وسیله­ی مدل بسیار دقیقی از اکسون مایلین­دار پستانداران انجام شد. تحریک الکتریکی یک فیبر عصبی شامل 51 گره رانویه با دو الکترود نقطه­ای یکی برای ایجاد بلوک و دیگری برای ایجاد پتانسیل عمل تست شبیه سازی شد و رفتار فیبر عصبی از جمله پدیده انسداد و مکانیزم تحقق آن مورد بررسی قرار گرفت. یافته ها: اعمال تحریک متناوب فرکانس بالا در حالت کلی سه رفتار متفاوت را در فیبر القا می­نماید. عدم پاسخ به تحریک، آتش شدن متناوب فیبر یا بلوک شدن فیبر پس از چندین بار آتش. با کاهش تقارن شکل­موج پیشنهادی دوفاز نامتقارن، آستانه لازم برای القای انسداد الکتریکی در فیبرهای عصبی کاهش می یابد. اعمال تحریک باعث افزایش متوسط ولتاژ غشاء می شود که می­تواند منجر به کاهش تحریک­پذیری غشاء و بلوک شدن فیبر گردد. نتیجه گیری: استفاده از شکل موج دوفاز نامتقارن در ایجاد انسداد عصبی سبب کارایی بالای این روش در بکارگیری بالینی می­شود. اعمال این شکل­موج باعث کاهش مصرف انرژی سیستم تحریک و همچنین کاهش آسیب به بافت شده و این مساله می­تواند گامی موثر در جهت استفاده بالینی از این روش برای کاهش درد، ویا ایجاد بی­حسی باشد.

Title: تأثیر برنامه آگاه سازی بر تغییر نگرش دانش آموزان دختر در زمینه جامعه پذیری و موقعیت تحصیلی نسبت به همسالان دارای اختلال جسمی – حرکتی در مدارس تلفیقی

http://jrrs.mui.ac.ir/index.php/jrrs/article/view/983

چکیده مقدمه: با افزایش تلفیق دانش آموزان دارای ناتوانی در کلاس‌های عادی، به کارگیری راهکارهایی که موجب بهبود نگرش نسبت به این افراد می‌شود، ضروری به نظر می رسد. هدف پژوهش حاضر بررسی تأثیر برنامه آگاه سازی بر بهبود نگرش دانش آموزان پایه پنجم ابتدایی در دو حیطه جامعه طلبی و موقعیت تحصیلی در مدارس تلفیقی نسبت به همسالان دارای ناتوانی جسمی- حرکتی است. مواد و روش ها: با استفاده از نمونه گیری چند مرحله ای 62 دانش آموز دختر پایه پنجم ابتدایی از 2 مدرسه تلفیقی شهر اراک در سال تحصیلی 92-1391 به صورت تصادفی انتخاب و در گروه مداخله (31نفر) و کنترل (31نفر) قرار گرفتند. با استفاده از پرسشنامه نگرش سنج گش (1993)، نگرش دانش آموزان مورد ارزیابی قرار گرفت. یافته ها، با استفاده از آزمون آماری تحلیل واریانس دو متغیری و آزمون توکی تجزیه و تحلیل شدند. یافته ها: نتایج بیانگر آن بود که نگرش دانش آموزان گروه مداخله، در مقیاس های جامعه طلبی و موقعیت تحصیلی، پس از شرکت در برنامه آگاه سازی در مقایسه با گروه گواه به طور معنادار افزایش یافته است. نتیجه گیری: شرکت دانش آموزان در برنامه آگاه سازی، موجب بهبود نگرش آن‌ها نسبت به همسالان دارای اختلال حسی- حرکتی می شود.

Title: تاثير برنامه تعادلی ستاره بر بازتواني آسيب اسپرين مچ پا

http://jrrs.mui.ac.ir/index.php/jrrs/article/view/741

چکیده مقدمه: اسپرین مچ پا از شايع ترين آسيب هاي حاد ورزشي است كه به علت آسيب به گيرنده هاي حس عمقي و كاهش تعادل، خطر بازگشت مجدد آن بالا است. هدف از تحقيق حاضر بررسي برنامه تمرین تعادلی ستاره بر بازتواني اسپرين مچ پا می باشد. مواد و روش‏ ها: تعدا 16 نفر از ورزشكاران غير حرفه اي مبتلا به اسپرين يك جانبه مچ پا در اين تحقيق شركت و يك دوره فيزيوتراپي را به مدت 12 جلسه سپري كردند. بعد از اطمينان از راه رفتن بدون درد، از آزمون تعادلي ستاره كه به‏عنوان يك برنامه بازتواني در درمان اسپرين مچ پا گزارش شده، به‏عنوان برنامه بازتواني به مدت 4 هفته استفاده و براي ارزيابي ميزان تاثير برنامه بازتواني از آزمون تعادلي ايستادن روي يك پا استفاده گرديد. ميزان آسيب مجدد بعد از يك سال ثبت گرديد. از آزمون تی وابسته براي تجزيه و تحليل داده‏ها استفاده شد. یافته ‏ها: نتايج تحقيق نشان داد كه برنامه تعادلی ستاره ای بر بازتوانی موثر بوده بگونه ای که بعد از دوره بازتواني، ميانگين نتايج آزمون تعادلي ايستادن روي يك پا بطور معنی‏داری بیشتر از دوره قبل از بازتوانی بود (001/0p < ) بعد از يك سال، 3 نفر از آزمودنی‏ها مجددا به اسپرين مچ پا مبتلا شدند. نتیجه گیری: با توجه به مثبت بودن برنامه تمرین تعادلی ستاره بر بازتوانی اسپرین مچ پا، این برنامه می تواند به‏عنوان پایه ای  در تسریع روند بهبودي و بازگشت مجدد آسيب قرار گیرد.

Title: مقایسه حافظه صریح و ضمنی کودکان با اختلال کم¬توجهی- بیش¬فعالی و همتایان عادی

http://jrrs.mui.ac.ir/index.php/jrrs/article/view/913

مقدمه: حافظه صریح بیش از حافظه ضمنی به منابع توجهی نیاز دارد. هدف این پژوهش مقایسه حافظه صریح و ضمنی کودکان مبتلا به اختلال کم­توجهی- بیش­فعالی و کودکان با تحول طبیعی است. مواد و روش­ها: پژوهش مقطعی مقایسه­ای در 15 دانش­آموز مبتلا به اختلال کم­توجهی- بیش­فعالی و 15 دانش­آموز سالم از مدارس شهرستان ابرکوه انجام شد. برای حافظه صریح لیست کلمات به فرد ارائه می­شد و فرد باید کلمات شنیده شده را یکبار بدون تأخیر و یک­بار با تأخیر بیان می­کرد. برای حافظه ضمنی نیز باید لغات شنیده شده را از بین لغات ارایه شده مشخص نماید. جهت تحلیل داده­ها از آزمون­های تی مستقل و تحلیل واریانس دو عاملی استفاده شد. یافته­ها: نتایج نشان داد که کودکان مبتلا به کم­توجهی- بیش­فعالی در حافظه صریح عملکرد پایین­تری نسبت به کودکان سالم دارند (05/0P<). همچنین در حافظه ضمنی، تفاوتی درعملکرد کودکان کم­توجه- بیش­فعال و سالم یافت نشد(05/0P>). نتیجه­گیری: کودکان با کم­توجهی- بیش­فعالی در حافظه صریح نقص دارند. پیشنهاد می­شود رویکردهای توانبخشی و آموزشی، حافظه ضمنی را برای مداخلات کودکان کم­توجه- بیش­فعال به کار گیرند.

Title: تاثیردوازده هفته تمرینات ادراکی- حرکتی بر تعادل پویای پسران کم¬توان ذهنی 11تا 14 سال

http://jrrs.mui.ac.ir/index.php/jrrs/article/view/1363

مقدمه: توجه به کنترل پاسچر در افراد کم­توان ذهنی به دلیل ضعف در اجرای وظایف تعادلی از اهمیت بالایی برخوردار می­باشد. هدف پژوهش حاضر ارزیابی اثربخشی دوازده هفته تمرینات ادراکی- حرکتی بر روی کنترل تعادل دینامیک دانش­آموزان پسر دارای ناتوانی ذهنی می­باشد. مواد و روش­ها: پژوهش حاضر از نوع  نیمه­تجربی و آزمایشگاهی می­باشد. 30 دانش آموز کم­توانی ذهنی با  بهره هوشی بین 55 تا 70 و میانگین سنی4/1±7/12 سال به صورت نمونه­گیری دردسترس انتخاب و در دو گروه کنترل و تجربی به طور تصادفی قرار گرفتند. قبل و بعد از تمرینات ادراکی- حرکتی تعادل دینامیک آزمودنی­ها توسط دستگاه بایودکس مورد سنجش قرار گرفت. دوره تمرینی 12 هفته، هفته­ای سه جلسه و هر جلسه به مدت 45 دقیقه بود. مولفه­های ادراکی حرکتی شامل: تعادل ایستا و پویا، شناسایی جهات مختلف، شناسایی بدن، ادراک فضا، حرکات هماهنگ و همزمان، ادراک بینایی، ادراک شنیداری، طرح حرکت، برتری جانبی، مهارت­های جابجایی و دستکاری بود. برای بررسی تفاوت بین پیش آزمون و پس آزمون از تست تی وابسته و برای مقایسه میزان شاخص­های تعادل و مقایسه شاخص­های تعادل در دو گروه طی پس­آزمون از آزمون آماری تحلیل کواریانس یک­سویه(ANCOVA)  با سطح معناداری 05/0 استفاده شد. نتایج: نتایج بیانگر بهتر بودن شاخص تعادلی قدامی- خلفی (003/0=P) و شاخص تعادلی کلی (017/0=P) به طور معنی­داری طی پس­آزمون در گروه تجربی در مقایسه با گروه کنترل بود. در حالی که شاخص داخلی-خارجی پیشرفت معناداری را نشان نداد (155/0=P). نتیجه­ گیری: تمرینات ادراکی- حرکتی استفاده شده در این پژوهش سبب بهبود تعادل، به ویژه در جهت قدامی- خلفی در آزمودنی­های مورد پژوهش گردید.

Title: بررسی ناهنجاری های قامتی و ارتباط آن با سن و جنسیت در دانش آموزان مقطع ابتدایی شهر تهران

http://jrrs.mui.ac.ir/index.php/jrrs/article/view/1091

مقدمه: شواهد پژوهشي نشان مي دهد كه ميزان ناهنجاري هاي قامتي به خصوص در افراد جوان، در حال افزايش است. ستون فقرات به عنوان محور اصلی بدن شناخته شده و نقش حیاتی آن حایز اهمیت است، چرا که هر گونه آسیب و تغییر شکل آن باعث اختلال در عملکرد بدن می گردد . هدف از اجراي این پژوهش بررسی ناهنجاري هاي قامتی و ارتباط آنها با سن و جنسیت در دانش آموزان ابتدایی شهر تهران می باشد مواد و روش ها: این مطالعه از نوع توصیفی-مقطعی بود که در آن 945 نفر از دانش آموزان مقاطع ابتدايی دخترانه و پسرانه مورد ارزيابي قرار گرفتند. براي انتخاب نمونه ها از روش نمونه گيري خوشه ای استفاده شد. در این مطالعه برای ارزیابی ناهنجاری پشت کج از تست Adam´s forward bend و برای ارزیابی کمر گود و پشت گرد از صفحه شطرنجی استفاده شد. تمامی اطلاعات به دست آمده توسط نرم افزار SPSS نسخه 18 مورد تجزیه و تحلیل قرار گرفت. یافته ها:نتایج نشان می دهد که شیوع ناهنجاری کمر گود در پسران 4/52% و در دختران 9/70%، پشت گرد در پسران 8% و در دختران 8/4% ،و پشت کج در پسران 8/0% و در دختران 3/1% می باشد.  همچنین میان شیوع ناهنجاری های کمر گود و پشت گرد با جنسیت و پایه تحصیلی دانش آموزان  همبستگی معناداری وجود داشت. نتیجه گیری: با توجه به نتایج به دست آمده، ضروري به نظر مي رسد كه با اجرای تست هاي غربالگری در كليه سطوح تحصيلی به ویژه مقطع ابتدایی نسبت به پيشگيري از بروز چنين ناهنجاري هايي در سنين بزرگسالی و همچنين درمان به موقع اقدامات لازم صورت گيرد.

Title: تبیین عوامل تسهیل کنندهپیروی والدین از برنامه آموزشی مراقبت ویژه ی کودکان فلج مغزی درمنزل: تحلیل محتوایی تجارب آموزشی والدین.

http://jrrs.mui.ac.ir/index.php/jrrs/article/view/1037

مقدمه: برنامه آموزشی مراقبت ویژه کودکان فلج مغزی، یکی از راه های مراقبتی- درمانی کودکان است. این برنامه با استفاده از اصول رویکرد عصبی- رشدی، قصددارد والدین را درجهت مراقبت و استقلال کودک خود توانمند سازد. به منظور اجرای موفقیت آمیزتر برنامه، کاردرمانگران باید عواملی که برتجربه پیروی والدین درمنزل اثر می گذارد را بررسی کنند. این مطالعه با هدف تبیین عوامل تسهیل کننده ارائه برنامه آموزشی (شرکت در یک کارگاه، دریافت کتابچه راهنما و پیگیری تلفنی در طول 3ماه) که براجرای والدین در مراقبت در منزل تاثیر گذاربوده، انجام گردید. مواد و روش ها: مطالعه از نوع تحلیل محتوایی کیفی بود. 15 مصاحبه عمیق با مادران کودکان فلج مغزی شرکت کننده در برنامه آموزشی مراقبت ویژه که سه ماه آن را درمنزل اجرا کرده بودند، انجام شد. مصاحبه ها ضبط شد وعینا به صورت دست نوشته هایی درآمد و به طریق استقرایی با رویکرد تحلیل محتوایی کانونشنال آنالیز شد. نتایج: عوامل تسهیل کننده ای که ازتجارب والدین بیرون آمدند، در سه طبقه اصلی "عوامل مربوط به کارگاه" ، "عوامل مربوط به کتابچه" و"ارتباط مداوم والدین با درمانگر کودک" جای گرفتند. بحث: عوامل متعددی استخراج شد که نشان می دهد، کاردرمانگران باید به  نحوه آموزش والدین کودکان فلج مغزی درمورد مسائل مراقبت ویژه توجه بیشتری داشته و در طول اجرای برنامه آموزشی مراقبت ویژه توسط والدین ارتباط حمایتگونه و مداوم خود را حفظ کنند.

Title: تفاوت های جنسیتی در فعالیت الکترومیوگرافی برخی از عضلات اندام تحتاني در افراد با و بدون ژنوواروم حین فرود بر روی یک پا

http://jrrs.mui.ac.ir/index.php/jrrs/article/view/1101

هدف: راستای نامناسب اندام تحتانی بویژه زانو، به دلیل تاثیر بر عملکرد عضلات و حس عمقی منجر به وقوع آسیب در این بخش می شود. از این رو هدف تحقیق حاضر بررسی تفاوت های جنسیتی در فعالیت الکترومیوگرافی برخی از عضلات اندام تحتاني در افراد دارای ژنو واروم با افراد سالم بود. مواد و روش ها: آزمودنی­های اين تحقيق 24 دانشجوی فعال (گروه یک: 6 زن دارای به ژنو واروم، گروه دو: 6 مرد دارای به ژنو واروم، گروه سه: 6 زن سالم، گروه چهار: 6 مرد سالم) دانشگاه تهران ، با دامنه سني 18 تا 22 سال بودند. براي جمع آوري داده ها، الكتوميوگرافي سطحي (EMG)، از عضلات گلوتئوس مدیوس، رکتوس فموریس، واستوس مدیالیس و تنسور فاشیالاتا انجام شده و ميزان فعالیت عضلات محاسبه شد. یافته ها: اختلاف بین گروهی معنی­دار در میزان فعالیت عضلات گلوتئوس مدیوس (001/0p=)، رکتوس فموریس (002/0p=) و واستوس مدیالیس (001/0p=) معنادار یافت شد اما اختلاف معنی­داری در میزان فعالیت عضله تنسور فاشیا لاتا (756/0p=) مشاهده نشد. نتایج آزمون تعقیبی بونفرونی تفاوت  معنی­داری در فعال سازی عضلات گلوتئوس مدیوس در زن (ژنو واروم)- مرد (ژنو واروم) (001/0p=)، راست رانی در زن (ژنو واروم)- مرد (ژنو واروم) (003/0p=)، واستوس مدیالیس در زن (زانو سالم)- زن (ژنو واروم) (003/0p=) و واستوس مدیالیس در زن (ژنو واروم)- مرد (ژنو واروم) (001/0p= نشان داد. نتیجه گیری: باتوجه به نتایج این تحقیق، نتیجه گیری می شود افراد دارای ناهنجاری ژنو واروم، مبتلا به نقص های کنترل نوروماسکولار هستند و این نقص­ها در زنان مبتلا به ژنو واروم بیشتر مردان است، از اینرو این افراد بیشتر از مردان در معرض خطر آسیب دیدگی قرار دارند.

Title: مقایسه استفاده از زمان در مادران دارای کودک مبتلا به اوتیسم و مادران دارای کودک عادی

http://jrrs.mui.ac.ir/index.php/jrrs/article/view/1304

مقدمه: آگاهی متخصصان توانبخشی و پزشکی، از چگونگی استفاده از زمان مادران دارای کودک مبتلا به اوتیسم می تواند به ارتقاء سطح سلامت مادر و خانواده کمک کند. مطالعات نشان داده که عموما مراقبت از کودکان کم توان به عهده مادر است و عملکرد مناسب مادر، به استفاده صحیح و مناسب از زمان، مرتبط می باشد. بنابراین در این پژوهش به بررسی چگونگی استفاده از زمان مادران دارای کودک مبتلا به اوتیسم پرداختیم. مواد و روش ها: در یک مطالعه از نوع مقطعی، نمونه گیری به روش در دسترس انجام شد. در هر گروه، 42 نفر مادر دارای کودک مبتلا به اوتیسم و 42 نفر مادر دارای کودک عادی شرکت کردند. از پرسشنامه دموگرافیک، آزمون گارز-2 و پرسشنامه استفاده از زمان مادران برای جمع آوری داده ها استفاده شد. داده ها با آزمون t مستقل و Chi- Square در SPSS17 تحلیل شدند. یافته ها: مادران دارای کودک مبتلا به اوتیسم در مقایسه با مادران دارای کودک عادی، در طی روز زمان بیشتری را صرف امور مربوط به کودک مبتلا می کردند (0/001>P)، اما در دیگر حیطه ها تفاوت معناداری بین دو گروه یافت نشد. همچنین کیفیت و لذت از فعالیتهای روزانه در مادران دارای کودک مبتلا به اوتیسم نسبت به مادران دارای کودک عادی بصورت معناداری پایین تر بود (P<0/05). اهمیت دادن به فعالیتهای روزانه برای مادران دو گروه تفاوت معناداری نداشت (P>0/05). نتیجه گیری: صرف زمان بیشتر مادران، برای امور مربوط به کودک مبتلا به اوتیسم، بر حیطه های فعالیتهای روزانه و همچنین جنبه های کیفی استفاده از ساعات روز تأثیر گذار است.

Title: بررسی حساسیت کنتراست رنگی در کودکان مبتلا به اختلال خواندن

http://jrrs.mui.ac.ir/index.php/jrrs/article/view/1174

مقدمه: اگرچه توافق جامعی در مورد اهمیت ادراک بینایی در فرآیند خواندن وجود دارد، نقش آن در ایجاد دیسلکسی شدیدا مورد بحث است. به منظور دریافتن ارتباط دیسلکسی با نقایص ادراک بینایی به مقایسه حساسیت کنتراست رنگی اپوننت در کودکان مبتلا به دیسلکسی و کودکان سالم پرداخته ایم. مواد و روش ها: 3 کودک دیسلکسیک و 3 کودک سالم در این مطاله شرکت داده شدند و پاسخ آنها به تحریکات ایجاد شده توسط نرم افزار Pattern Generator در سه فرکانس فضایی 5,8,18cpd ثبت شد. آزمون حساسیت کنتراست در هر فرکانس فضایی در دو مرحله تشخیص ظاهر شدن خطوط(pattern detection) و درک تمایز رنگ (color discrimination) در دو زمینه زرد و آبی انجام گرفت. یافته ها: در مجموع تفاوت بین افراد نرمال و دیسلکسیک قابل توجه نبود و در اغلب موارد تفاوت معنا­داری بین این دو گروه دیده نشد. با این حال افراد دیسلکسیک چه در زمینه زرد و چه در زمینه آبی در تست تشخیص ظاهر شدن خطوط حساسیت کاهش یافته ای را نشان دادند اما در آزمون درک تمایز رنگ نسبت به گروه کنترل حساسیت بهتری داشتند. نتیجه گیری: نتایج حاصل از مقایسه افراد دیسلکسیک و گروه کنترل نشان داد حساسیت کنتراست رنگی شاخص تمایز دهنده مناسبی برای این دو گروه نیست. بنابراین به نظر می رسد در بیماران دیسلکسیک تمامی خصوصیات عملکردی سیستم بینایی متاثر نشده است. همچنین این امکان وجود دارد که در بیماران دیسلکسیک با وجود طبیعی بودن پاسخ های پایه و اولیه بینایی، پاسخ های ادراکی متفاوتی نسبت به افراد نرمال داشته باشیم.

Title: مروري بر ويژگي هاي پتانسيل هاي عضلاني برانگيخته دهليزي با تأكيد بر پاسخ گردني

http://jrrs.mui.ac.ir/index.php/jrrs/article/view/1437

مقدمه:ظهور نسبتاً جديد آزمون VEMP گردني و متعاقب آن VEMP چشمي، عرصه جديدي از ارزيابي سيستم وستيبولار را فرا روي شنوايي شناسان و محققان سيستم شنوايي وستيبولي قرار داده است. با توجه به مسير عصبي خاصي كه در طي ثبت اين پتاسيل مورد ارزيابي قرار مي گيرد مي توان آن را به عنوان آزموني مكمل، جهت بررسي سيستم تعادلي تلقي كرد. هدف اين مطالعه فراهم كردن مروري از آخرين اطلاعات درباره ي روش هاي مختلف استفاده شده براي ثبت پتانسيل عضلاني برانگيخته دهليزي و بررسي  تغيير اين پتانسيل در بيماري هاي مختلف درگير كننده ي سيستم وستيبولار بود. مواد و روش ها: جستجو بين مقالات 10 سال اخير با كليد واژه ي پتانسيل عضلاني برانگيخته دهليزي در بانك هاي اطلاعاتيGoogle Scholar،PubMed ،Ebsco ، Science Direct و همچنين كتب مرجع مرتبط با موضوع انجام شد. يافته ها: از 58 مقاله براي بسط و توضيح مفاهيم اصلي و روش هاي استفاده شده براي انجام VEMP استفاده شد. نتيجه گيري: با انجام صحيح آزمونVEMP و حذف عوامل مخدوشگر تأثير گذار بر پاسخ، و با تكيه بر نتايج تحقيقات معتبر در اين زمينه مي توان از آن به عنوان ابزار تشخيصي مهمي در ارزيابي بيماري هاي مختلف درگير كننده ي وستيبول و بررسي روند درمان اين بيماري ها بهره گرفت.
